# A Web-Based Platform for People With Memory Problems and Their Caregivers (CAREGIVERSPRO-MMD): Mixed-Methods Evaluation of Usability

**DOI:** 10.2196/formative.9083

**Published:** 2018-03-12

**Authors:** Paraskevi Zafeiridi, Kevin Paulson, Rosie Dunn, Emma Wolverson, Caroline White, Jonathan Adrian Thorpe, Marco Antomarini, Francesca Cesaroni, Francesca Scocchera, Isabelle Landrin-Dutot, Laëtitia Malherbe, Hendi Lingiah, Marie Bérard, Xavier Gironès, Maria Quintana, Ulises Cortés, Cristian Barrué, Atia Cortés, Ioannis Paliokas, Konstantinos Votis, Dimitrios Tzovaras

**Affiliations:** ^1^ School of Engineering and Computer Science University of Hull Hull United Kingdom; ^2^ School of Health and Social Work University of Hull Hull United Kingdom; ^3^ Cooperativa Sociale Onlus Marche Onlus Ancona Italy; ^4^ Department of Internal Medicine, Geriatrics and Therapeutics Rouen University Hospital Rouen Cedex France; ^5^ Department of Geriatrics Rouen University Hospital Rouen Cedex France; ^6^ Faculty of Health Sciences University of Vic Central University of Catalonia Manresa Spain; ^7^ Knowledge Engineering & Machine Learning Group Universitat Politècnica de Catalunya Barcelona Spain; ^8^ Information Technologies Institute Centre for Research and Technology Hellas Thessaloniki Greece

**Keywords:** dementia, technology, social support, caregivers

## Abstract

**Background:**

The increasing number of people with dementia (PwD) drives research exploring Web-based support interventions to provide effective care for larger populations. In this concept, a Web-based platform (CAREGIVERSPRO-MMD, 620911) was designed to (1) improve the quality of life for PwD, (2) reduce caregiver burden, (3) reduce the financial costs for care, and (4) reduce administration time for health and social care professionals.

**Objective:**

The objective of this study was to evaluate the usability and usefulness of CAREGIVERSPRO-MMD platform for PwD or mild cognitive impairment (MCI), informal caregivers, and health and social care professionals with respect to a wider strategy followed by the project to enhance the user-centered approach. A secondary aim of the study was to collect recommendations to improve the platform before the future pilot study.

**Methods:**

A mixed methods design was employed for recruiting PwD or MCI (N=24), informal caregivers (N=24), and professionals (N=10). Participants were asked to rate their satisfaction, the perceived usefulness, and ease of use of each function of the platform. Qualitative questions about the improvement of the platform were asked when participants provided low scores for a function. Testing occurred at baseline and 1 week after participants used the platform. The dropout rate from baseline to the follow-up was approximately 10% (6/58).

**Results:**

After 1 week of platform use, the system was useful for 90% (20.75/23) of the caregivers and for 89% (5.36/6) of the professionals. When users responded to more than 1 question per platform function, the mean of satisfied users per function was calculated. These user groups also provided positive evaluations for the ease of use (caregivers: 82%, 18.75/23; professionals: 97%, 5.82/6) and their satisfaction with the platform (caregivers: 79%, 18.08/23; professionals: 73%, 4.36/6). Ratings from PwD were lower than the other groups for usefulness (57%, 13/23), ease of use (41%, 9.4/23), and overall satisfaction (47%, 11/23) with the platform (*P*<.05). Qualitative comments related to both improvements for functionality and the platform interface.

**Conclusions:**

Although caregivers and professionals were overall satisfied with the platform, further adaptations were recommended by PwD. This reiterates the importance of the involvement of end users in the development of Web-based interventions. Recommendations from users in this paper apply for the interface and functionality of a wider range of Web-based support interventions.

## Introduction

### Background

The increasing number of people with dementia (PwD) and its progressive nature has led researchers to explore tools to provide support to larger numbers of PwD and their caregivers. A range of Web-based support interventions have been designed and evaluated including websites providing material and training for caregivers [1,2] and Web-based portals to enable communication with health care professionals [3]. Other Web-based support interventions combine educational material, communication with health care professionals, and monitoring of PwD well-being through Web-based questionnaires [4].

To aid the development of successful Web-based support interventions, researchers utilize user-centered designs to refine devices and technology to meet the needs of the targeted population [5]. A continuous and iterative involvement of users (eg, through focus groups or interviews) is widely seen vital in the design of technological solutions [6]. Usability in this context is measured as the user-friendliness (eg, ease to learn) and perceived usefulness in addressing users’ needs [7].

In a recent study, a Web-based portal (the Digital Alzheimer Center; DAC) was developed in Netherlands for PwD and caregivers [8]. DAC provides information on dementia, promotes peer support and communication, and enables communication with health professionals. The usefulness and usability of the DAC was assessed through evaluation from PwD and caregivers. Both the participant groups found DAC useful. Involving users in the development and evaluation of Web-based support interventions enables researchers to understand their unmet needs and increase user autonomy [9]. Other projects developing technological devices for PwD and caregivers have also taken into account the perceived usability from the perspective of end users. In the Skills Training and Reskilling (STAR) project [3], a Web-based training portal was developed to offer learning opportunities to caregivers, as well as peer support and contact with care professionals. Informal caregivers, volunteers in dementia care, and professional caregivers rated the STAR Web-based portal as useful and user-friendly. In the Rosetta project [10], 3 previously developed tools were merged, the COGKNOW Day Navigator [11], the Emerge system [12], and the Unattended Autonomous Surveillance system. This platform is offered through a touch screen and provides reminders for activities, a phone dialing system with pictures, a radio button, activity support for performing everyday tasks (eg, preparing coffee), and safety warnings (eg, the door is open). The platform also offers monitoring and emergency function with sensors monitoring daily activities, as well as automatic detection of emergencies. Data from PwD, informal caregivers, professionals, and dementia experts were collected to rank the usefulness of the Rosetta functions and to collect information about improving the system.

### Aims and Objectives

This study aims to explore the usability and user-friendliness of the CAREGIVERSPRO-MMD platform [13] through evaluations performed by PwD or mild cognitive impairment (MCI), informal caregivers, and health and social care professionals. The CAREGIVERSPRO-MMD platform targets the dyad of PwD or MCI and their informal caregivers, alongside their health and social care professionals. The platform is being developed based on a user-centered approach identifying (1) the characteristics of PwD and caregivers affecting their ability to use Web-based tools and (2) the user requirements for the platform functionality. The platform is being piloted at 4 centers in Italy, Spain, France, and the United Kingdom. One innovation of the CAREGIVERSPRO-MMD platform is that it integrates many features that have previously been tested individually, namely: (1) peer-to-peer social contacts through circles of friends, (2) forums or cafes for open discussions, (3) practical information about dementia and local resources, (4) open monitoring of user well-being through Web-based questionnaires and activity measures through interactions with the platform, and (5) guided personalized educational material about living with dementia or MCI and caregiving. The platform integrates a gamification engine designed to increase user engagement. Behind the platform, a machine learning engine will attempt to present the features of the platform to users to maximize the benefit. The aim of the platform is to improve the quality of life for PwD or MCI and reduce caregiver stress. Secondary aims are to delay institutionalization for PwD, reduce care costs, and reduce administration time and costs for professionals. The functions available in the early version of the platform in the usability test are presented in Table 1.

**Table 1:** Platform functions tested in this usability study.

Name	Activity of platform functions
Home page	Sharing and replying to messages
My Network	Creating a network of peers
My profile	Uploading personal information
My Agenda	Recording appointments and events and setting reminders
Invitations	Reviewing invitations sent from other users
Café (Forum)	Sharing information, tips, and support in a social networking forum
My Health	Completing Web-based questionnaires to monitor health and well-being
Local resources	Seeking information about local services that offer help and support
Create user profiles	Creating accounts to enroll people with dementia (PwD) and caregivers to the platform (for professionals only)
Managed users	Reviewing the profiles of PwD^a^and caregivers (for professionals only)

^a^PwD: people with dementia.

The aim of this study was to test the usability of the early version of the platform (usefulness, ease of use, user satisfaction) for PwD or MCI, primary caregivers and professionals. A secondary aim was to generate recommendations from users that could be utilized to further improve the platform.

## Methods

### Design

The study employed a mixed methods design and included the collection of both quantitative and qualitative data. This is in line with the previous studies that have employed a combination of qualitative and quantitative methods to measure the usability of Web-based support interventions. In the COGKNOW project [7], data for the user-friendliness, the usefulness, and the effectiveness of the intervention were collected though qualitative interviews and questionnaires. In a similar way, the researchers in the DAC project [8] collected usability data through observation, a Web-based survey, and semistructured interviews. The mixed methods designs combine the benefits from both quantitative and qualitative approaches and increase the validity of results [14]. This is because the mixed method designs capture the understanding of participants for a topic or a concept through closed, quantitative questions and provide a deeper understanding for the responses of participants through open, qualitative questions. Data for mixed methods designs can be collected through a questionnaire including both closed and open questions [15,16]. Therefore, the participants in this study completed questionnaires with both closed questions about the perceived ease of use, usefulness and their satisfaction with each function of the platform, and with open questions in the event that participants were not satisfied with one or more of the platform functions.

**Figure 1:**
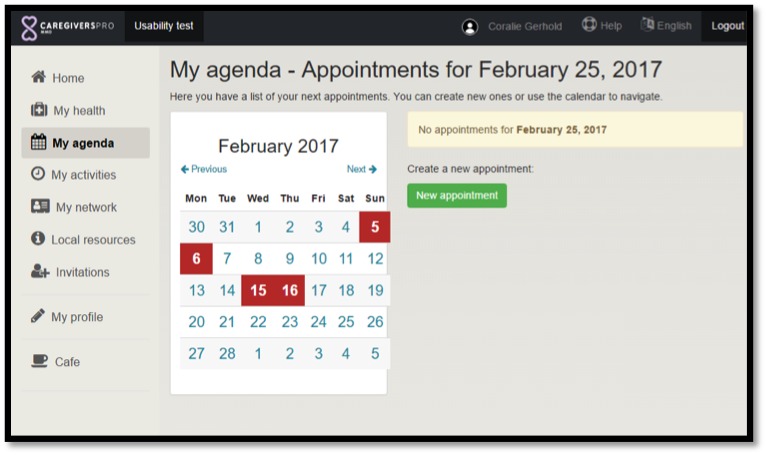
The "My Agenda" function.

**Figure 2:**
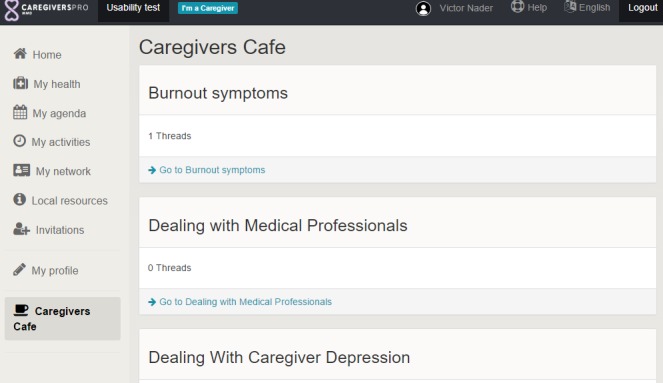
The "Café" function.

Following a convergent parallel design [14], the researchers in this study collected quantitative and qualitative data simultaneously and then merged both types of data to interpret the results. Examples of platform functions are presented in Figures 1 and 2. Ethical approval for CAREGIVERSPRO-MMD project is obtained from the Ethics committee of the Faculty of Health and Social Care (United Kingdom), the Comitato Etico Regionale delle Marche (Italy), the Centre Hospitalier Universitaire de Rouen (France), the Comité de Protection des Personnes (France), the Fundació Universitària del Bages (Spain), and the Comité de Ética de Investigación Clínica Fundació Unió (Spain).

### Participants and Recruitment

Users were recruited in Ancona (Italy), Hull (United Kingdom), Manresa (Spain), and Rouen (France) from local health and social care providers and community support groups. Inclusion criteria for PwD or MCI (N=24) were (1) to have a self-reported diagnosis of dementia or MCI, (2) to be at least 50 years old, and (3) to have an informal primary caregiver who agreed to participate too. All PwD or MCI were retired from work. For primary caregivers (N=24) and professionals (N=10), the inclusion criteria required them to be older than 18 years and have adequate language skills in the country of testing, and for the caregivers to be an informal, unpaid carer supporting PwD or MCI. A total of 12 caregivers were employed on a full-time basis in addition to their caregiving responsibilities. Another 12 caregivers were retired from work. Demographic characteristics are presented in Table 2.

**Table 2:** Demographic characteristics.

Characteristic		Value
**Age in years, mean (SD, age range)**		
	PwD^a^/MCI^b^(n=24)	78.30 (9.70, 55-91)
	Caregivers (n=24)	53.58 (13.71, 30-77)
	Professionals (n=10)	40.78 (10.44, 26-53)
**PwD/MCI gender, n (%)**		
	Males	10 (41.67)
	Females	14 (58.33)
**Level of education for PwD/MCI, n (%)**		
	No qualifications after school	9 (37.5)
	Higher education qualifications	12 (50)
	Other training (ie, vocational)	3 (12.5)
**Diagnosis for PwD/MCI, n (%)**		
	Alzheimer’s disease	14 (58.33)
	Mixed dementia (Alzheimer's disease and vascular dementia)	3 (12.5)
	MCI	3 (12.5)
	Vascular dementia	1 (4.17)
	Other	3 (12.5)
**Years living with the diagnosis, n (%)**		
	Up to 5 years	8 (33.33)
	5-10 years	2 (8.33)
	More than 10 years	5 (20.83)
	Unknown	9 (37.5)
**Caregivers gender, n (%)**		
	Males	4 (16.67)
	Females	20 (83.33)
**Level of education for caregivers, n (%)**		
	No qualifications after school	8 (33.33)
	Higher education qualifications	16 (66.66)
	Other training (ie, vocational)	0 (0)
**Caregivers relationship with PwD, n (%)**		
	Spouses	9 (37.5)
	Children	11 (45.83)
	Grandchildren	1 (4.17)
	Other relatives	3 (12.5)
**Hours of caregiving per week, n (%)**		
	2-14 hours	9 (37.5)
	15-25 hours	1 (4.17)
	40 hours	1 (4.17)
	56-168 hours	7 (29.17)
	Unknown	6 (25)
**Professionals gender, n (%)**		
	Males	3 (30)
	Females	7 (70)
**Professionals job role, n (%)**		
	Doctors (neurologists and geriatricians)	5 (50)
	Psychologists	3 (30)
	Social workers	1 (10)
	Nurses	1 (10)

^a^PwD: people with dementia.

^b^MCI: mild cognitive impairment.

### Materials

All testing material was developed initially in English and subsequently translated by the researchers of the project into the other languages (French, Italian, and Spanish). These materials included printed information sheets for participants, consent forms, demographic sheets, usability questionnaires for each user group (PwD/MCI, informal caregivers, health and social care professionals), and a short user guide for the platform.

A usability questionnaire was developed for each user group based on the different platform functions designed for each group. The questionnaires for PwD and caregivers consisted of 30 items, and the questionnaire for professionals included 15 items. The questionnaires were developed based on usability questions from the previous research [3,7] and from questions emerging from the previous stages of the project. Thus, the questionnaires included questions about the ease of use and usefulness of each platform function, as well as about user satisfaction. They were designed to be administered by researchers. The questionnaires also included questions about the willingness of users to use the platform in the future and to recommend it to others. Responses were recorded on a 5-point Likert scale from 0 to 4 indicating strong disagreement to strong agreement. When users provided a neutral or negative score (2 or less on the Likert scale), they were asked by researchers to provide further information and suggestions for the improvement of the platform function.

### Procedure

Once users consented to participate in the study, researchers created individual accounts and demonstrated the platform. During the demonstration of each platform function, users were verbally asked to rate the ease of use, usefulness, and their satisfaction with the platform. When users provided a neutral or negative response to the quantitative questions, they were immediately asked to provide qualitative feedback about this function. This method was used to avoid confusion, that is, by asking feedback for all platform functions together. Scores were collected by researchers through the usability questionnaires at baseline and the 1-week follow-up to measure the usability of the platform before and after users had access to it for 1 week. Participants were tested at the Rouen University Hospital (France), at the Sant Andreu Hospital of the Sociosanitari Foundation of Manresa (Spain), at the Centro Diurno Anziani *Licio Visintini* (Italy), or at their own environment (United Kingdom). Technical support was available via phone or home visits.

### Data Analyses

Demographics were analyzed with descriptive statistics. Quantitative data from the questionnaires were analyzed with nonparametric tests to show differences in the perceived usefulness and usability of the platform between baseline and follow-up testing, and differences between the user groups.

Data from qualitative questions were analyzed with thematic analysis [17]. PZ, EW, CW, and RD read and reread the interview transcripts and identified the emerging themes. The themes were discussed until consensus was reached and are presented in the Results section. Qualitative data aim to support the quantitative findings and to provide a deeper understanding of the quantitative responses from participants. Therefore, quantitative and qualitative data are merged for interpretation in the Discussion section [14].

## Results

For analysis, the mean and percentages of users within each group, who agreed with the statements supporting the platform functions (responding 3 or 4 on the Likert scales), are presented in Table 3. When users were asked more than 1 question per platform function, the mean of satisfied users was calculated. Of all users, 6 users (1 PwD, 1 informal caregiver, 4 professionals) participated only at baseline testing and were excluded from further analysis.

At baseline, the platform was considered useful by the majority of PwD (65%, 15.5/24), informal caregivers (87%, 21/24), and professionals (85%, 8.45/10). Satisfaction rates were also positive from most of PwD (58%, 13.92/24), caregivers (83%, 20/24), and professionals (65%, 6.45/10). Ease of use scores did not follow the same pattern, with 48% (11.58/24) for PwD finding the platform easy to use. In contrast, 85% (20/24) of caregivers and 84% (8.36/10) of professionals appreciated the ease of use of the platform.

Scores from the follow-up indicated that the perceived usefulness, ease of use, and satisfaction with the platform increased for professionals after using the platform for 1 week. The ease of use and user satisfaction declined for PwD and caregivers, as well as in perceived usefulness for PwD.

### Usability Scores at Baseline and Follow-Up Visits

To enable comparisons between the usability scores at baseline and after 1 week of platform use, the means and standard deviations of usefulness, ease of use, and satisfaction for all platform functions were calculated for each user group (Table 4).

Mann-Whitney *U* tests did not confirm significant differences between baseline and follow-up usability scores for PwD, caregivers, or professionals.

Tables 3 and 4 reveal a discrepancy between the baseline and follow-up scores for PwD. Although usefulness means are increased for PwD at the follow-up compared with the baseline (Table 4), fewer PwD find the platform useful (Table 3).

### Usability Scores Across the Three User Groups

Kruskal-Wallis *H* tests revealed significant differences at baseline between PwD, caregivers, and professionals in usefulness (H(2)=12.1, *P*=.01), ease of use (H(2)=14.4, *P*<.001), and satisfaction (H(2)=12.1, *P*=.01). Post hoc tests showed these differences to be between PwD and carers in usefulness (*P*<.001), ease of use (*P*<.001), and satisfaction (*P*<.001). Differences were also found between PwD and professionals in the ease of use (*P*=.02).

The results from the follow-up data also revealed significant differences between the user groups in usefulness (H(2) =16.6, *P*<.001), ease of use (H(2)=18.5, *P*<.001), and satisfaction (H(2)=12.0, *P*=.01). These differences were found between PwD and carers in usefulness (*P*<.001), ease of use (*P*<.001), and satisfaction (*P*<.001). Differences were also found between PwD and professionals in usefulness (*P*=.01), ease of use (*P*<.001), and satisfaction (*P*=.045).

### Usability for Platform Functions and Suggestions for Improvement: Overall Feedback and Guidance for Technology Projects

#### Interface

PwD and caregivers preferred bigger color contrasts and font sizes, as well as images and icons rather than text menus. Emoticons were used in the platform to like or not-like messages, but both PwD and caregivers found this confusing.

**Table 3:** Number of users and percentages (in parentheses) agreeing with the usability of CAREGIVERSPRO-MMD platform functions.

Usability variables	PwD^a^/MCI^b^, n (%)		Caregivers, n (%)		Professionals, n (%)	
	Baseline	1-week follow-up	Baseline	1-week follow-up	Baseline	1-week follow-up
Usefulness	15.5 (65)	13 (57)	21 (87)	20.75 (90)	8.45 (85)	5.36 (89)
Ease of use	11.58 (48)	9.4 (41)	20 (85)	18.75 (82)	8.36 (84)	5.82 (97)
Satisfaction	13.92 (58)	11 (47)	20 (83)	18.08 (79)	6.45 (65)	4.36 (73)

^a^PwD: people with dementia.

^b^MCI: mild cognitive impairment.

**Table 4:** Baseline versus follow-up usability scores for all user groups.

User group/usability variable		Baseline, mean (SD)	Follow-up, mean (SD)
**PwD^a^/MCI^b^**				
	Usefulness	2.40 (1.03)	2.43 (1.09)
	Ease of use	2.09 (1.20)	2.08 (1.11)
	Satisfaction	2.34 (1.00)	2.22 (1.07)
**Caregivers**				
	Usefulness	3.22 (0.55)	3.39 (0.48)
	Ease of use	3.16 (0.83)	3.16 (0.88)
	Satisfaction	3.18 (0.67)	3.17 (0.70)
**Professionals**				
	Usefulness	3.13 (0.43)	3.44 (0.53)
	Ease of use	3.13 (0.60)	3.59 (0.27)
	Satisfaction	2.85 (0.61)	3.18 (0.70)

^a^PwD: people with dementia.

^b^MCI: mild cognitive impairment.

#### Additional Functions

PwD suggested including cognitive training games in the platform to train their memory. Caregivers wanted an easy way to send instant messages to health and social care professionals.

#### Language Used

Caregivers commented on the language used on the platform. They felt that terms such as “dementia” should be avoided in favor of *memory problems*.

#### Privacy

All users were concerned with the privacy of information that PwD and caregivers insert in the platform. They suggested using short explanations in each page of the platform to remind users who will see each piece of information.

### Feedback per Platform Function

#### Home

The major social-network function of the platform allows users to publish messages to circles of friends and to reply to messages. At baseline there was wide acceptance that these functions would be useful and had been implemented in a way that made them easy. For caregivers and professionals, this perception either remained the same or was reinforced by use of the platform. However, PwD were less convinced and this did not increase with experience (Tables 5 and 6). PwD found it difficult to find previous published messages and pictures.

#### My Network

The My Network feature allows users to establish their circle of friends with whom they can share information and posts. This process involves the sending and possible acceptance of an invitation. PwD rated this feature lower than the other user groups, especially for the ease of use. Caregivers would prefer to receive notifications about invitation requests to the PwD they care for. Caregivers suggested that no notification was better than a reject notification, if an invitation was not accepted, to avoid upsetting users. They also suggested they would like to be able to find new contacts through common interests such as hobbies. Professionals required more information about PwD and the ability to download a file containing all the uploaded information and the responses to questionnaires by PwD.

#### My Profile

PwD provided the lowest usability scores and satisfaction rates among the 3 user groups for updating personal information. PwD and caregivers were concerned about privacy settings and who had access to the information they uploaded.

#### My Agenda

Caregivers and professionals rated higher, than PwD, the usability of the agenda function for noting appointments and events. All user groups reported that they would like to control who can see their own appointments. PwD and caregivers also suggested that the upcoming appointments should be presented in a chronological order (with the closest appointment on the top of the page). PwD also suggested having options for selecting which appointments are displayed, for example, appointments could be selected to be displayed on a monthly, weekly, or daily basis.

#### Invitations

The majority of caregivers and professionals appreciated reviewing invitations from other users. PwD would prefer a standout notification, such as a notification in red color. Caregivers would prefer to be able to click on notifications to read them, as well as to show notifications in the home page to alert them for a request.

#### Café (Forum)

The forum was rated lower by PwD than by other user groups. The majority of PwD expressed their concerns about a possible inappropriate use and the need for the forum to be monitored by administrators. PwD and caregivers also suggested that they needed information about who can see information in the forums and the need to keep the individual forums for PwD, caregivers, and professionals separate. However, in contrast, professionals suggested that their user group should have access to all forums.

#### My Health

This platform function for uploading health and medical information for PwD and caregivers appears as *Manage users* to professionals and was the least appreciated of all the functions. Professionals reported that important information about PwD, including their cognitive level, is missing (see Create User Profiles and Manage Users). PwD underlined the need to include only user-friendly questionnaires with general questions, such as generic questions about their mood. PwD and caregivers requested a record of scores from completed questionnaires. Caregivers were concerned about who can see their information.

#### Local Resources

The majority of people in all user groups expressed overall satisfaction with the information provided about local resources. This platform function was the most appreciated function by PwD and caregivers. PwD and caregivers suggested a comment box under each local resource to leave feedback for other users should be available. Caregivers suggested that users should be able to upload new local resources.

#### Create User Profiles and Manage Users

Scores from professionals indicated their satisfaction with creating profiles for users, while they gave a lower rating for the platform function for monitoring PwD and caregivers. Professionals suggested that scores for PwD cognitive ability should be provided. They also suggested a “user summary” to be generated for them, including information about the health and current emotional well-being of PwD and caregivers.

**Table 5:** Number of satisfied people with dementia (PwD), mild cognitive impairment (MCI), and caregivers for each platform function, excluding the sign in function.

Platform functions		Usefulness (mean)		Ease of Use (mean)		Satisfaction (mean)	
		Baseline (N=24)	1-week follow-up (N=23)	Baseline (N=24)	1-week follow-up (N=23)	Baseline (N=24)	1-week follow-up (N=23)
**Home**							
	PwD^a^/MCI^b^	17	17	14	13	19	17
	Caregivers	21	22	19	18	19	19
**My Network**							
	PwD/MCI	14.5	12	12.5	9.5	14	10
	Caregivers	20.5	21.5	21	18.5	21	18
**My Profile**							
	PwD/MCI	14	12	11	8	13	9.5
	Caregivers	20	20.5	20	18.5	21	18
**My Agenda**							
	PwD/MCI	14.5	13	11.5	10	12	10.5
	Caregivers	19.5	18.5	20.5	17.5	19	16
**Invitations**							
	PwD/MCI	17	14	10	8	15	11
	Caregivers	21	21	19	17	18	17
**Café (Forum)**							
	PwD/MCI	15	12	9	7	10	9
	Caregivers	22	21	22	22	16	16
**My Health**							
	PwD/MCI	18	12	11	10	16	11
	Caregivers	21	20	20	19	21	18
**Local Resources**							
	PwD/MCI	21	17	11	10	16	12
	Caregivers	24	23	22	21	24	21

^a^PwD: people with dementia.

^b^MCI: mild cognitive impairment.

**Table 6:** Number of satisfied professionals for each platform function, excluding the sign in function.

Platform functions	Usefulness (mean)		Ease of Use (mean)		Satisfaction (mean)	
	Baseline (n=10)	1-week follow-up (n=6)	Baseline (n=10)	1-week follow-up (n=6)	Baseline (n=10)	1-week follow-up (n=6)
Home	7	5	9	6	6	5
My Network	9.5	5.5	8	5.5	5.5	3.5
My Profile	8	6	8	5.5	3.5	2.5
My Agenda	9.5	6	8.5	6	8.5	6
Invitations	9	6	9	6	8	6
Create User Profiles	10	6	10	6	9	5
Managed Users	7	3	6	6	4	3
Café (Forum)	7	4	9	6	9	5

## Discussion

### Principal Findings

This study explored the usability of the current version of the CAREGIVERSPRO-MMD platform through ratings and feedback provided from PwD or MCI, primary caregivers, and health and social care professionals. The results revealed significant differences in the usability scores of the 3 user groups, with caregivers and professionals rating the platform more useful and easy to use than PwD. Differences in scores between baseline and 1 week after using the platform were not statistically significant. However, although the mean of perceived usefulness of the platform for PwD was increased in the follow-up testing, the number of users finding the platform useful was decreased. Analysis of the individual functions of the platform showed that the 3 user groups held different opinions of the usability of the platform functions. Professionals considered the function to connect with users in the platform for peer support a necessary function and providing information about themselves as professionals to be less useful. PwD and caregivers considered the information for local resources to be the most important function of the platform for them and the peer support forum to be the least important. PwD appreciated some functions of the platform, such as the social networking service, and showed their interest to communicate with others; however, their scores for the ease of use of these functions underline their inexperience with technology. Suggestions for further platform functions for PwD and MCI concern games for cognitive training and instant communication with health and social care professionals through the platform. Professionals suggested including important health information about PwD and caregivers that is currently missing. These findings show that priorities differ between the 3 user groups and thus, platforms for each user group should be designed to fit the needs of each particular group. Findings from this study can be used for the development of future Web-based interventions, as well as for the further development of CAREGIVERSPRO-MMD platform.

### Developing Web-Based Interventions Based on User Characteristics

PwD evaluated the platform as less useful and easy to use than caregivers and professionals. This difference may reflect the age difference between the mean ages of the user groups since PwD were 20 to 40 years older than caregivers and professionals. This finding is in line with the literature [18], where older adults needed more time than young adults to perform tasks using touch screens. The decline of cognitive functioning for PwD and MCI may be another explanation for the low usability scores. Literature suggests that PwD prefer less cluttered webpages, with less information per page, requiring less cognitive effort than other user groups [8].

Another possible explanation of the variability in the evaluation of the 3 groups concerns their experience with technology. The majority of PwD reported no previous knowledge of accessing the Web. Evidence suggest that older adults are keen to use technology devices when they are trained to use them [19], when they are aware of the benefits [20], when technology enables their communication with other people, and when they have previous experience with computers at work [21].

The discrepancies between the usability scores of the 3 user groups can also be explained by the different needs of these groups. The lower scores of PwD indicate the need to adapt the interface and functionality of the platform to meet their needs, such as to simplify the interface for this group. The need to adjust the interface according to personal preferences is in line with the previous research [10] as individual preferences vary.

### Guidance for Technology Projects

Qualitative results from this study may act as a guide for developing future Web-based support interventions for PwD or MCI and their caregivers.

### Interface

The need of PwD and caregivers for less busy pages in the platform, more images, larger font size and color contrasts, and fewer colors on each page shows the importance of platform adaptation and adjustment for each user group. The technology design for older people needs to be adjusted to their motor, sensory, and cognitive abilities, including their visual and auditory capacity [6] because age-related impairments are likely to affect older adults’ engagement with computer systems [18].

### Content and Functionality

PwD and caregivers were concerned about the privacy of the platform. They requested explanations about who has access to their information and underlined the importance of monitoring the platform for an inappropriate use.

The variability in the importance of each platform function between the user groups, such as PwD finding Web-based questionnaires not useful but professionals needing more questionnaires, suggests that the dyads of PwD and caregivers have different needs and interests than those which may be anticipated by professionals and developers. This finding underlines the importance of involving end users in the development of Web-based support interventions to meet their needs [9].

The main limitation of this study was the lack of privacy and security arrangements in the early version of the platform. Future projects should consider the suggestions provided by PwD and caregivers in this study when developing technological interventions. Simpler interventions can be developed for PwD with uncluttered interfaces and an appropriate number of functionalities so that end users will engage with the interventions. Privacy issues about sharing information on Internet can be addressed with short statements explaining who can see this information while implementing in the platform all the regulations related with data protection and privacy at the European and national levels. Future research on technology-based platforms can also collect data about the usage of the platform. In a similar way, data for the number of visits per platform page could show the preferences of users.

### Conclusions

Involving end users in the development of Web-based support interventions is necessary to understand their needs and preferences. The discrepancies in the evaluations from PwD, caregivers, and professionals highlighted that these needs and preferences vary in each group. The different preferences have been identified both in respect of the interface and the content of the platform. The feedback collected through this study will not only inform the development of CAREGIVERSPRO-MMD platform but also provide valuable suggestions for the development of Web-based support interventions for PwD and caregivers.

## Abbreviations

DAC: Digital Alzheimer Center
MCI: mild cognitive impairment
PwD: people with dementia
STAR: Skills Training and Reskilling
